# Development and Evaluation of a Semi-Nested PCR Method Based on the *18S ribosomal RNA* Gene for the Detection of *Babesia aktasi* Infections in Goats

**DOI:** 10.3390/vetsci11100466

**Published:** 2024-10-01

**Authors:** Mehmet Can Ulucesme, Sezayi Ozubek, Munir Aktas

**Affiliations:** Department of Parasitology, Faculty of Veterinary Medicine, University of Fırat, Elazığ 23200, Türkiye; mculucesme@firat.edu.tr (M.C.U.); sozubek@firat.edu.tr (S.O.)

**Keywords:** *Babesia aktasi*, goats, semi-nested PCR, specific primers, *18S ribosomal RNA* gene

## Abstract

**Simple Summary:**

We developed a new test to detect *Babesia aktasi*, a parasite that infects goats, using a method called semi-nested PCR. This method focuses on a specific part of the parasite’s DNA to ensure accuracy. We checked the test against several other similar parasites to make sure it only detected *B. aktasi*, which it did successfully. To see how sensitive our test is, we used blood samples with known amounts of the parasite and found that our test could detect even very low levels of infection. Our results show that this new test is both highly accurate and sensitive, making it a valuable tool for identifying *B. aktasi* infections in goats. This new PCR method provides a reliable tool for detecting *B. aktasi* in goats, which is crucial for managing and preventing the spread of this infection, ultimately protecting goat health and improving agricultural productivity.

**Abstract:**

We developed and evaluated a semi-nested PCR assay for the detection of *Babesia aktasi* infection in goats based on the sequence of the *B. aktasi 18S ribosomal RNA* gene. Following in silico screening, the specificity of the primers was assessed using reference DNA samples, including *B. ovis*, *B. motasi*, *B. crassa*, *B. venatorum*, *B. divergens*, *B. capreoli*, *Theileria ovis*, and *T. annulata*. To determine the sensitivity of the method, blood infected with 2% parasitemia of *B. aktasi* was diluted to 10-fold serial dilutions. The method specifically amplified a 438 bp fragment of *B. aktasi* DNA, but did not demonstrate cross-amplification with the other hemoparasites tested. The sensitivity assay indicated that this PCR method was able to detect infection at a dilution of 10^−8^ of 2% parasitemia (0.074 parasites/200 µL). Ninety-seven blood samples collected from goats were used to analyze for *B. aktasi*, and the infection was detected in 18.5% of the goats. Additionally, the method was also applied to 44 field DNA samples that were detected to be positive for *B. aktasi* by reverse line blotting (RLB), and showed 84.1% agreement. The findings revealed that newly developed semi-nested PCR can detect *B. aktasi* infections in goats with high sensitivity and specificity.

## 1. Introduction

*Babesia* species infect a diverse array of vertebrate hosts, including domestic and wild hosts, humans, and birds, in tropical and subtropical regions worldwide [1,2,3]. These parasites have a significant economic impact on the livestock industry due to losses from animal deaths and the costs of preventive control measures [3,4,5,6]. Babesiosis is marked by severe clinical illness (high fever, anemia, jaundice, and hemoglobinuria), potentially resulting in death in hosts [5,7,8,9,10]. The disease, transmitted by ixodid ticks, was first described in cattle and sheep in 1888 [11]. While bovine babesiosis has been extensively investigated worldwide, there is still a notable gap in knowledge regarding small ruminant babesiosis. Developments in cell biology and molecular parasitology have led to new insights into ovine babesiosis and discovery of novel pathogens in sheep and goats, including *Babesia* sp. Xinjiang, *Babesia lengau*-like, and variants of *B. motasi*-like [3,12,13,14]. Recently, a novel *Babesia* sp., named *Babesia aktasi*, was described from an indigenous goat in Turkey, expanding the known *Babesia* fauna in small ruminants [15,16]. Caprine babesiosis caused by *B. aktasi* can manifest as subclinical infections in non-immunosuppressed indigenous goats, but it can cause severe clinical infections resulting in deaths in immunosuppressed individuals [17].

Microscopy is still the most cost-effective and suitable method for the detection of the intra-erythrocytic forms of the parasite in acute babesiosis [18]. However, due to the morphological similarity of *Babesia* species, this method is insufficient for species identification [19,20]. While microscopic examination is considered the gold standard for diagnosing babesiosis, its sensitivity can be limited in cases of low parasitemia, particularly in atypical or chronic infections, where parasite levels may fall below the detection threshold of microscopy [20]. Serology is often used to determine the prevalence of small ruminant babesiosis in epidemiological studies, but their specificity is limited due to cross-reactions with other *Babesia* and *Theileria* parasites [21,22]. These methods can also yield false-positive and -negative results, and they cannot distinguish between past exposure and current infections [21,23]. Therefore, it is important to develop molecular diagnostic methods for the identification of *Babesia* infections at the species level (for specific and sensitive detection of the parasite DNA). Indeed, advances in molecular parasitology have made molecular diagnostic tools the preferred methods for diagnosis of hemoprotozoan parasites, as the techniques offer greater sensitivity and specificity compared to microscopy and serology for detection of *Babesia* infections [24,25].

Several molecular techniques have been developed and applied for the diagnosis of *Babesia* infections in small ruminants, including conventional polymerase chain reaction (PCR) [4,26,27,28], semi-nested PCR [29], multiplex PCR [30], real-time PCR [31], loop-mediated isothermal amplification (LAMP) [32], cross-priming amplification combined with a vertical flow [33], and reverse line blot hybridization (RLB) assays [24,34,35,36,37,38,39]. These methods have significantly enhanced the sensitivity and specificity of the diagnosis. Among them, the RLB assay, which combines PCR with a blotting procedure, can simultaneously detect and identify *Babesia*, *Theileria*, *Anaplasma*, and *Ehrlichia* species in a single sample [40,41]. The assay was previously used as a screening tool in our laboratory for epidemiological surveys [36,38,42]; however, it is labor-intensive and has a slow turnaround.

In the current study, multiple sequence alignment based on the V4 hypervariable region of the *18S ribosomal RNA* (*18S rRNA*) gene, which is conserved in all *Babesia* and *Theileria* parasites, was performed using the sequence of *B. aktasi*, three ovine *Babesia* species commonly found in sheep (*B. ovis*, *B. motasi*, and *B. crassa*), and other *Babesia* species (*B. venatorum*, *B. odocoilei*, *B. divergens*, and *B. capreoli*) that showed close similarity to the *B. aktasi* sequence (MN559399) in a BLAST search. In this study, the results were utilized to design a semi-nested PCR assay aimed at detecting *B. aktasi* infections in goats. The specificity, sensitivity, and field applicability of this method were evaluated. The assay was used to analyze 97 blood samples collected from apparently healthy goats for the presence of *B. aktasi* DNA. Additionally, 44 archived DNA samples, previously identified as *B. aktasi*-positive via the reverse line blot (RLB) assay [42], were reanalyzed, and the results were compared to evaluate the consistency of detection between the two methods. These findings provide a basis for the development of a reliable diagnostic tool for *B. aktasi* in both clinical and research settings

## 2. Materials and Methods

### 2.1. Ethics Statement

This study was conducted in compliance with the regulations of Turkish legislation for animal protection and welfare. This work was reviewed and approved by the Fırat University Animal Experiments Local Ethics Committee (research clearance 2021/12). Written informed consent was secured from the animal owners for their participation in this study.

### 2.2. Primer Design

*Babesia* and *Theileria* parasites have a V4 hypervariable region of the *18S rRNA* gene. Extensive sequence variation in this region provides species differentiation among these tick-borne hemoparasites [40,43]. In this study, three oligonucleotide primers were designed for a semi-nested PCR targeting a 438 bp fragment of the V4 hypervariable region of the *18S rRNA* gene of *B. aktasi* (GenBank accession number MN559399). The nucleotide sequences of *Babesia* parasites commonly found in small ruminants and indicating close similarity to the MN559399 sequence of *B. aktasi* by BLAST search were aligned using Multiple Alignment with Fast Fourier Transform (MAFFT) version 7 [44] to pinpoint unique regions specific to *B. aktasi* (Supplementary Figure S1). The alignments were analyzed to identify sequence variations and to check the semi-nested primer positions for any mismatches with the *B. aktasi* sequence. These mismatches could potentially hinder annealing, amplification, and detection of species-specific DNA. The GenBank accession numbers used the MAFFT analysis for the primer design were as follows: MN559399 (*B. aktasi*), AY998123 (*B. ovis*), AY260179 (*B. motasi*), AY260176 (*B. crassa*), GQ888709 (*B. venatorum*), KC460321 (*B. odocoilei*), KP745627 (*B. divergens*), and KP742785 (*B. capreoli*).

### 2.3. Standard Positive Reference DNA Samples Used in This Study

In our previous study, *B. aktasi* was isolated from a naturally infected indigenous goat [16]. In the current study, genomic DNA extracted from this stabilate was used as a reference positive control (MN559399) for the development of *B. aktasi*-specific semi-nested PCR. Plasmid DNA of *B. motasi* was kindly provided by Dr. Ana Hurtado from the Department of Animal Health, Instituto Vasco de Investigacion y Desarrollo Agrario (NEIKER) Berreaga, Spain. Plasmid DNAs of *B. venatorum* and *B. capreoli* were kindly provided by Professor Martin Pfeffer from Epidemiologie Biochemie, Universitat Leipzig, Germany. DNAs of *B. ovis* (EF092454), *B. crassa* (KF034782), *B. divergens* (KP745627), *T. ovis* (EF092452), and *T. annulata* (AY508463) were obtained from cattle, sheep, and ticks in previous studies and stored in our laboratory. These positive reference DNA samples were utilized to determine the specificity of semi-nested PCR assays. The negative blood sample was collected from a 1-month-old goat that had been confirmed negative for *Babesia* spp., *Theileria* spp., and *Anaplasma* spp. by molecular techniques.

### 2.4. Semi-Nested PCR Amplification of 18S rRNA Gene

All reference DNA samples were subjected to semi-nested PCR using the designed external and internal primers to amplify target 438 bp fragments of V4 hypervariable region of the *18S rRNA* gene. For the first round of PCR, 2.5 µL 10X PCR buffer (VitaTaq, Procomcure Biotech, Australia), 2.5 µL of each dNTP at 1.25 mM, 0.1 μL of *Taq* DNA polymerase (5 U/μL), 0.5 μL each of outer and inner primers (20 pmol/μL), 2.5 μL of template DNA, and 16.4 μL of ddH_2_O were used in 25 µL of final volume for the reactions. One microliter of the amplicon from the first-round PCR was used as the template in semi-nested PCR. Reactions were conducted in an automated DNA Sensequest thermal cycler (Labcycler Gradient, Göttingen, Germany). Touchdown PCR and thermal cycling conditions were carried out as previously reported [38]. Briefly, the cycling conditions were denaturation for 5 min at 94 °C, followed by 94 °C for 20 s, 67 °C for 20 s, and 72 °C for 30 s. The annealing temperature was decreased every second cycle with 2 °C to a “touchdown” temperature of 57 °C. The amplicons were analyzed using electrophoresis on 1.5% agarose gels, and the expected band pattern was visualized using the Quantum Vilber Lourmat (France) gel imaging system.

### 2.5. Efficiency and Detection Threshold of the Semi-Nested PCR

For assay specificity, the nucleic acids of *B. aktasi* as well as other tick-borne hemoparasites such as *B. ovis*, *B. motasi*, *B. crassa*, *B. venatorum*, *B. divergens*, *B. capreoli*, *T. ovis*, and *T. annulata* were used as other reference templates for the PCR amplification. Goat blood DNA negative for *Babesia* spp., *Theileria* spp., and *Anaplasma* spp. was used as a negative control, as well as PCR-grade water in each PCR reaction.

*Babesia aktasi*-infected blood with 2% parasitemia obtained from the experimentally infected goat was used to assess the efficiency and detecting threshold of the semi-nested PCR assay. The infected blood was diluted to 10-fold serial dilutions (from 10^−1^ to 10^−10^) with the non-infected goat blood to calibrate the parasite concentrations from 7.4 × 10^−2^ (parasitemia: 0.0000000002%) to 7.4 × 10^7^ (parasitemia: 2%) infected erythrocytes/200 µL of the total erythrocytes [25,26,45]. Genomic DNA was isolated from 200 µL of each diluted sample using a commercial DNA isolation kit (PureLinkTM Genomic DNA Mini Kit, Invitrogen Corporation, Carlsbad, CA, USA) according to the custom protocol. The samples were amplified by the semi-nested PCR using specific primers for *B. aktasi* DNA. Thus, the minimum detectable parasitemia was determined by observing agarose gel results of semi-nested PCR amplicons.

### 2.6. Field Application of Semi-Nested PCR in Genomic DNAs Isolated from Goats

A total of 97 whole-blood samples were collected from the jugular vein of the apparently healthy indigenous goats into 5 mL vacuum tubes with EDTA in breeding farms located in the villages of Anamur district in Mersin Province, Turkey where *B. aktasi* is known to be endemic [42]. The DNA extraction was made from 200 µL blood samples using the PureLink™ Genomic DNA Mini Kit (Invitrogen Corporation, Carlsbad, CA, USA) according to the kit guide. The DNAs were used as template in the semi-nested PCR for the evidence of *18S rRNA* gene of *B. aktasi.* The PCR reactions were conducted in a 25 µL volume, as described in Section 2.4. To confirm the semi-nested PCR amplifications, 3 representative positive products obtained from field samples were then purified and subjected to Sanger DNA sequencing using the newly designed forward primer. In addition, 44 archived DNA samples that tested positive for *B. aktasi* by nested PCR-based RLB in our recent field survey [42] were also screened for the presence of the parasite in the current study.

## 3. Results

### 3.1. Primer Selection for Semi-Nested PCR

**Figure 1:**
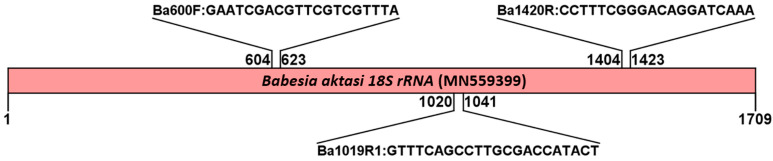
Schematic representation of the DNA-binding regions for the primers Ba600F, Ba1420R, and Ba1019R1 selected for the detection of *B. aktasi* by semi-nested PCR assay. The primer selection was based on multiple alignment results using the *B. aktasi 18S rRNA* gene sequence (MN559399) and various *Babesia* sequences registered in GenBank.

The alignments of *18S rRNA* gene sequences of various *Babesia* species showed that there was a high degree of nucleotide variation, particularly at the 5′ end in the V4 region of *B. aktasi 18S rRNA* gene. Three primers species-specific to *B. aktasi* were designed, including one forward primer (Ba600F) and two reverse primers (Ba1420R and Ba1019R1). A schematic representation of the DNA-binding regions of candidate nucleotide sequences selected based on the multiple alignment results for PCR amplification of *B. aktasi* DNA is shown in Figure 1.

The designed primers determined to be specific to *B. aktasi* in silico were tested using *Babesia*- and *Theileria*-positive reference DNA samples. Additionally, field DNA samples obtained from asymptomatic goats were screened for the presence of *B. aktasi*. The details of the designed primers (primer name, nucleotide sequence, reaction and/or use, and amplicon size) are presented in Table 1.

### 3.2. Evaluation of Analytical Specificity of the Semi-Nested PCR

**Figure 2:**
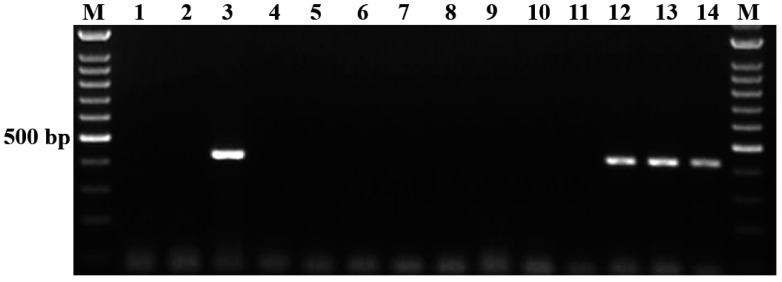
Specificity of semi-nested PCR. Agarose gel electrophoresis of semi-nested PCR products (438 bp) from *Babesia*- and *Theileria*-positive reference controls and field DNA samples using *B. aktasi*-specific primers. M, 100 bp marker; lines 1 and 2, negative controls (1, PCR-grade water; 2, genomic DNA obtained from a one-month-old goat not infected with *Babesia*, *Theileria*, or *Anaplasma* species); lines 3–11, standard positive-control DNA samples (3, *B. aktasi*; 4, *B. ovis*; 5, *B. motasi*; 6, *B. crassa*; 7, *B. divergens*; 8, *B. venatorum*; 9, *B. capreoli*; 10, *T. ovis*; 11, *T. annulata*); lines 12–14, field DNA samples collected from apparently healthy goats infected with *B. aktasi*.

**Figure 3:**
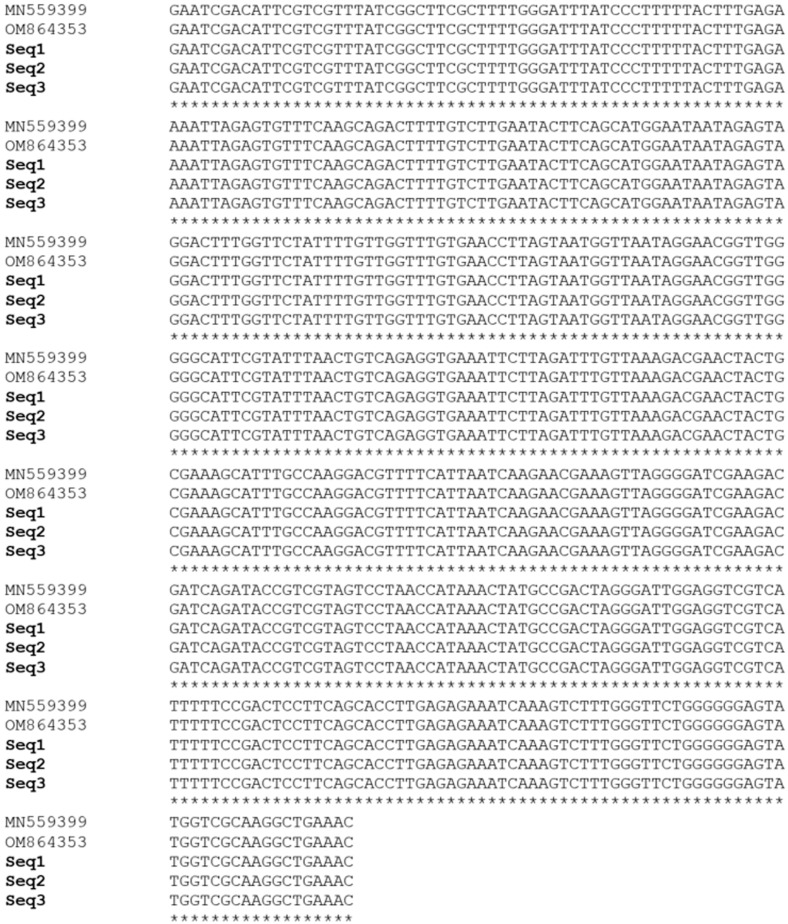
Multiple sequence alignment of three *B. aktasi* sequences (Seq1–3) obtained from the semi-nested PCR assay aligned with *B. aktasi* reference sequences (MN559399 and OM864353) available in GenBank.

The results of the specificity assay obtained in this study are presented in Figure 2. Following the gel electrophoresis of the amplicons, the species-specific primers to *B. aktasi* successfully generated single and clear positive amplicons of 438 bp for the *18S rRNA* gene exclusively from *B. aktasi*-positive reference DNA (Figure 2, lines 3). However, no positive amplification products were detected in other *Babesia* (*B. ovis*, *B. motasi*, *B. crassa*, *B. divergens*, *B. venatorum*, *B. capreoli*)- or *Theileria* (*T. ovis*, *T. annulata*)-positive reference DNA samples (Figure 2, lines 4–11), suggesting that the primers are specific for the detection of *B. aktasi*. The single and clear bands were also detected in some field samples collected from goats (Figure 2, lines 12, 13, 14). To verify the results obtained from the field samples, three randomly selected positive amplicons were sequenced (Seq1–3). The BLAST analysis results of the obtained sequences showed 100% similarity with the previously published *B. aktasi* sequences MN559399 and OM864353 (Figure 3). Thus, the in silico analysis results were confirmed by the laboratory findings, and it was verified that the selected primers were specific to *B. aktasi*.

### 3.3. Analytical Sensitivity of the Semi-Nested PCR

**Figure 4:**
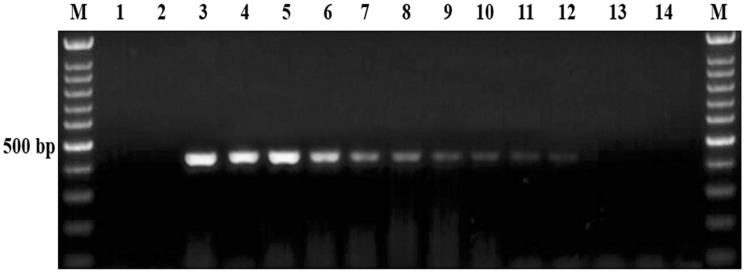
Sensitivity of semi-nested PCR. Agarose gel image of the PCR amplicons from DNA isolated from 10-fold serial dilutions (10^−1^ to 10^−10^) of infected blood containing 2% parasitemia. M, 100 bp marker; lanes 1–2, standard negative controls (1, PCR-grade water; 2, genomic DNA obtained from a one-month-old goat not infected with *Babesia*, *Theileria*, or *Anaplasma* species); 3, positive control (*B. aktasi*, GenBank accession number MN559399); 4, *B. aktasi* DNA isolated from 2% parasitic blood; lanes 5–14, DNA samples isolated from 10-fold dilution series ranging from 10^−1^ to 10^−10^.

To evaluate the sensitivity of the semi-nested PCR method, *B. aktasi* DNA extracted from 10-fold serial dilutions of blood containing 2% parasitemia was amplified using newly developed primers, and the results were presented in Figure 4. Agarose gel electrophoresis of the amplified products revealed differences in detection limits between the initial PCR and the semi-nested PCR assays. The initial PCR detected the presence of the target at a dilution of 10^−5^, while the semi-nested PCR demonstrated a significantly higher sensitivity, detecting the target at a dilution of 10^−8^. In terms of infected erythrocyte detection limits, the initial PCR was able to identify 7.4 × 10^1^ infected erythrocytes per 200 µL. In contrast, the semi-nested PCR displayed a higher sensitivity, with a detection limit of 7.4 × 10^−2^ infected erythrocytes per 200 µL (equivalent to 0.074 parasites per 200 µL).

### 3.4. Field Application and Detecting Performance of Semi-Nested PCR on Field Blood Samples

DNA samples extracted from field blood samples collected from goats were analyzed using the semi-nested PCR method to demonstrate the field utility of this method as a diagnostic tool for epidemiological studies. Out of 97 blood samples collected from asymptomatic goats in the region where the parasite is endemic, 18 were found to be infected with *B. aktasi*, indicating a high prevalence of 18.5% (18/97). To confirm the PCR results, three representative positive amplicons were sequenced by Sanger DNA sequencing and confirmed for *B. aktasi*. These amplicons were found to be 99% to 100% homologous to *B. aktasi* (GenBank accession number MN559399).

To validate and evaluate the performance of semi-nested PCR assays, 44 DNA samples that tested positive with the RLB for *B. aktasi* was tested for the parasite DNA in the current study. *Babesia aktasi* was amplified in 37 out of 44 DNA samples. This result revealed that 84.1% (37/44) agreement was observed between the semi-nested PCR and the RLB. All these findings revealed that the newly developed semi-nested PCR in this study can be an effective molecular diagnostic tool in subsequent molecular surveys for specific and highly sensitive detection of *B. aktasi* infection in goats.

## 4. Discussion

In this study, the *18S rRNA* gene sequence of *B. aktasi* was aligned with other *Babesia* sequences available in GenBank. Based on this alignment, three primers were designed for the detection of *B. aktasi*. Using these species-specific primers, reference positive *Babesia* and *Theileria* DNAs were subjected to amplification with the semi-nested PCR. The newly designed primers showed specificity consistent with that of the *B. aktasi 18S rRNA* gene ion semi-nested PCR. Additionally, we reported the detection threshold of the method for identifying *B. aktasi*.

Small ruminant babesiosis is a tick-borne hemoparasitic disease caused by the protozoan parasites of the genus *Babesia*. The disease is widespread in tropical and temperate regions worldwide, where ixodid tick vectors are widespread [4,9,46,47,48]. These parasites are primarily transmitted by ticks of the genus *Rhipicephalus* and invade red blood cells, leading to symptoms such as fever, anemia, jaundice, and in severe cases, death [5,9,10]. The disease poses a considerable economic threat to small-ruminant production due to decreased productivity, treatment costs, and mortality [7]. Effective control and prevention strategies include tick management, use of acaricides, and chemical treatment of infected individuals. The primary pathogens responsible for babesiosis in small ruminants include *B. ovis*, *B. motasi*, and *B. crassa* [3,12,15,49,50]. Molecular studies over the past two decades in small ruminants have also discovered novel *Babesia* species or genotypes, such as *B. aktasi*, *Babesia* sp. Xinjiang, *Babesia lengau*-like, and *B. motasi*-like variants, indicating a greater genetic diversity of small-ruminant *Babesia* lineage than previously understood [3,12,14,35,42]. These newly discovered species or genotypes suggest the need for ongoing surveillance and developing and updated diagnostic methods to effectively manage and control of babesiosis in small ruminants. Diagnosis of the disease is typically achieved through blood smear examination, serological tests, and PCR-based molecular methods.

The conventional technique for diagnosing *Babesia* infections in vertebrate hosts relies on examining thin blood smears under a light microscope. However, this technique can be challenging in carrier hosts with low level of parasites and even in acute cases at the early stages of the disease. Additionally, microscopy requires a high level of expertise, as *Babesia* piroplasms have similar morphology, which can lead to confusion, especially in cases of co-infection [51,52,53]. Serology has also been used, but can encounter issues with specificity and sensitivity [20,22]. Rapid and precise identification of tick-borne pathogens is fundamental to understand their epidemiology. Therefore, molecular methods, particularly PCR techniques that target the *18S rRNA* gene, are effective for detecting and identifying small-ruminant *Babesia*/*Theileria* parasites [24,26,36]. These PCR-based methods address the challenges associated with detecting low levels of parasitemia and facilitated species identification. *Babesia aktasi* has recently been identified as a novel *Babesia* species in indigenous goats in Turkey [15,16]. Our recent large-scale molecular survey carried out in indigenous goats revealed that the parasite is quite prevalent in some parts of the country [42]. Experimental infection studies have reported that *B. aktasi* causes fatal clinical infections in immunosuppressed goats [17]. However, there is no PCR method available for the diagnosis of *B. aktasi*. Here, a highly specific and sensitive semi-nested PCR assay for the detection of *B. aktasi* infections in goats was developed by designing species-specific primers targeting a 438 bp fragment within the V4 hypervariable region of the *18S ribosomal RNA* gene.

Evaluation of the semi-nested PCR using the designed primers demonstrated the ability of this method to amplify *B. aktasi* and produce a clear band without cross-reacting with *B. ovis*, *B. motasi*, *B. crassa*, *B. venatorum*, *B. divergens*, *B. capreoli*, *T. ovis*, or *T. annulata* (Figure 2). This indicates that the assay is specific for the detection of *B. aktasi* infections in goats. The primers successfully detected *B. aktasi* infection in field samples collected from asymptomatic goats as well. By applying the semi-nested PCR test to 97 blood samples collected from the locations where the parasite is prevalent, we found that *B. aktasi* had a high detection rate of 18.5% (18/97) in field samples. The proportion of *B. aktasi*-positive goats was consistent with a previous survey reported by Ulucesme et al. [42] from the Mediterranean region of Turkey (22.5%) using the RLB assay. The specificity of the test was also confirmed by the fact that the DNA sequences of three representative amplicons obtained from field samples showed 100% similarity to the *B. aktasi 18S rRNA* gene sequences reported in GenBank. In addition, the performance of the semi-nested PCR assay in detecting *B. aktasi* was tested on 44 archived DNA samples previously confirmed as *B. aktasi*-positive by the RLB assay. The results indicated an 84.1% (37/44) agreement between the semi-nested PCR and RLB assays. This strong agreement underscores the consistency and reliability of the semi-nested PCR assay developed in this study. However, the RLB assay is likely more sensitive than the semi-nested PCR, which may explain the failure to detect *B. aktasi* in seven of the samples. The RLB assay’s ability to detect low parasitemia or degraded DNA through hybridization techniques gives it an edge in sensitivity over PCR-based methods, which could account for the discrepancy. Despite this minor difference, these findings indicate that the newly developed semi-nested PCR assay can be reliably and effectively used as a screening tool in molecular epidemiological studies. It provides a robust alternative for laboratories that may not have access to RLB assay kits while maintaining a high level of accuracy for detecting *B. aktasi*. One limitation of the current study is that only RLB-positive samples were included for the evaluation of the semi-nested PCR assay. As a result, we were able to assess the sensitivity of the assay, but not to evaluate its specificity or calculate agreement metrics such as the kappa coefficient. Future studies that incorporate both RLB-positive and RLB-negative samples will be essential to provide a more comprehensive assessment of the assay’s diagnostic performance, including specificity, sensitivity, and overall agreement with other diagnostic tools.

The detection limit of diagnostic tests is crucial for accurate and timely pathogen identification. This parameter determines the smallest pathogen quantity reliably detected, impacting test sensitivity and effectiveness [54]. It has been noted that in dogs, *Babesia gibsoni* infections with low levels of parasitemia (ranging from 0% to 0.75%) can sometimes be misdiagnosed as immune-mediated hemolytic anemia [55]. In this study, the sensitivity assay performed for the detection of *B. aktasi* demonstrated that the developed semi-nested PCR assay has high sensitivity. Using this assay, it is possible to detect 7.4 × 10^−2^ infected erythrocytes (0.074 parasites/200 µL). Our results indicated that the semi-nested PCR can be used to detect *B. aktasi* at even very low parasitemia levels in goats. Similar sensitivity has been reported for *B. ovis* [26,56], *T. annulata* [57], *B. bovis* [45,58], *B. equi* and *B. caballi* [25,53], *B. rossi* and *B. vogeli* [59], and *B. bigemina* [60]. In field conditions, a subset of animals may exist in a carrier state, where they do not exhibit obvious symptoms of disease. These carrier animals act as reservoirs of infection, potentially transmitting the disease to naïve ticks. Furthermore, their movement can introduce the disease to new areas, posing a risk of spreading the infection to previously unaffected regions. Identifying the carrier hosts is crucial, as they play a significant role in the epidemiology of tick-borne hemoparasitic pathogens.

## 5. Conclusions

In conclusion, we developed a novel semi-nested PCR method for the detection of *B. aktasi*. The specificity and sensitivity of the assay were assessed and evaluated for the detection of the parasite in goat blood. Additionally, the newly designed primers were tested on field samples collected from goats. The assay provides a useful and applicable diagnostic tool for detecting subclinical cases and monitoring carrier animals. Furthermore, this study has expanded our current knowledge about the occurrence of goat babesiosis caused by *B. aktasi*.

## Figures and Tables

**Table 1:** Primers designed in this study for *B. aktasi* DNA amplification.

Gene	Primer Name	Primer Sequences (5’→3’)	Reaction and/or Use	Amplicon Size
*18S rRNA*	Ba600F Ba1420R	GAATCGACGTTCGTCGTTTA CCTTTCGGGACAGGATCAAA	First round PCR forward F First round PCR reverse R	820
	Ba600F Ba1019R1	GAATCGACGTTCGTCGTTTA GTTTCAGCCTTGCGACCATACT	Semi-nested PCR forward F Semi-nested PCR reverse R1	438

## Data Availability

The raw data supporting the conclusions of this article will be made available by the authors on request.

## Supplementary Materials

The following supporting information can be downloaded at https://www.mdpi.com/article/10.3390/vetsci11100466/s1. Figure S1: Nucleotide sequence alignment of the target regions of *18S ribosomal RNA* genes. Sequences marked in yellow indicate primer Ba600F, sequences marked in blue indicate primer Ba1019R1, and sequences marked in green indicate primer Ba1420R.
