# Supplementary figures and images for: Development and Evaluation of a Semi-Nested PCR Method Based on the 18S ribosomal RNA Gene for the Detection of Babesia aktasi Infections in Goats
Source: Vet Sci. 2024 Oct 1;11(10):466. doi: 10.3390/vetsci11100466 (PMC11511400; doi:10.3390/vetsci11100466)

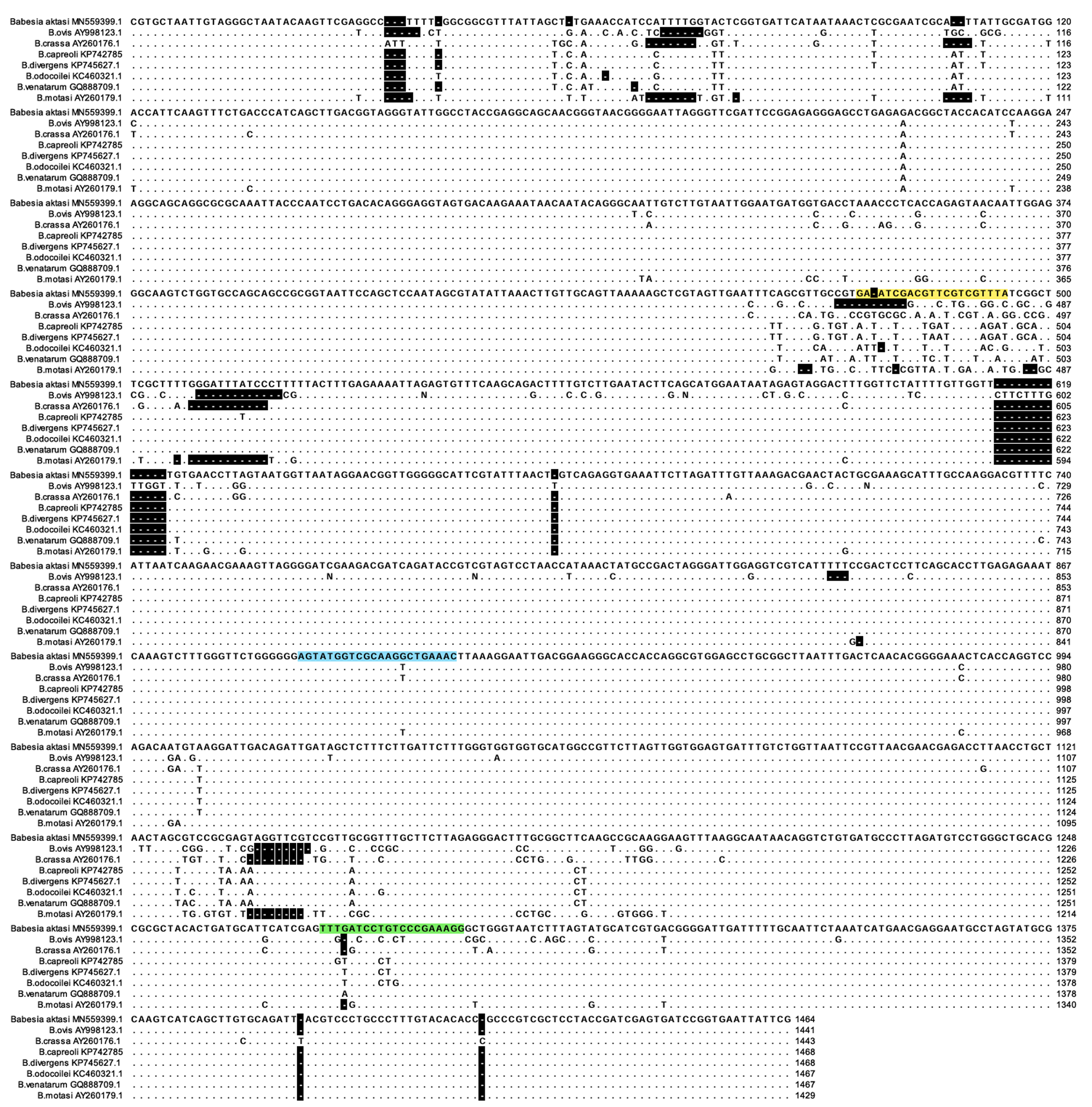

Supplement: Supplementary file 1 [file vetsci-11-00466-s001.zip › vetsci-3168751-supplementary.tif]
